# VX-765 has a Protective Effect on Mice with Ovarian Injury Caused by Chemotherapy

**DOI:** 10.2174/1568009622666220930110024

**Published:** 2023-01-11

**Authors:** Pingyin Lee, Canquan Zhou, Xiaokun Hu

**Affiliations:** 1 Reproductive Medicine Center, The First Affiliated Hospital of Sun Yat-sen University, Guangzhou, China;; 2 Guangdong Provincial Key Laboratory of Reproductive Medicine, The First Affiliated Hospital of Sun Yat-sen University, Guangzhou, China

**Keywords:** VX-765, cyclophosphamide, ovarian injury, ovarian protection, PI3K / PTEN / AKT pathway, burn out

## Abstract

**Background:**

Malignant tumors continue to remain a main global public health issue. In the past 40 years, due to strides made in multi-disciplinary comprehensive treatment schemes for patients suffering from malignant tumors, especially chemotherapy schemes, the survival rate has been greatly improved in such patients. This group can be expected to maintain their fertility or have restored endocrine function following successful malignant tumor treatment. Therefore, focusing on the ovarian damage caused by chemotherapy in women of childbearing age is vital in order to protect their fertility and improve their quality of life.

**Objective:**

This study attempted to evaluate whether VX-765 possesses an ovarian protective effect in ovarian injury induced by chemotherapy in the mice model.

**Methods:**

Female C57BL/6J mice were administered with VX-765 gavage once a day for 21 consecutive days. Use of cyclophosphamide (Cy) began one week after the last gavage administration of VX-765. Detailed classification of follicles at various levels was then quantified in each group. Immunohistochemistry and Western blot analysis were then used in order to analyze the expression of key proteins (FOXO3a, mTOR, RPS6 and AKT) as well as their phosphorylation of the PI3K / PTEN / AKT pathways in the ovary. The concentrations of AMH were measured by ELISA.

**Results:**

The follicles at all levels of Cy treated mice were less than those of the normal group (*P* < 0.05). Meanwhile, mice treated with VX-765 prior to receiving Cy treatment had more primordial follicles (PMF) than mice treated with Cy alone (*P* < 0.05). In early growing follicles (EGF) and antral follicles (AF), no difference was observed among the experimental groups (*P >* 0.05), however, they were lower than those in the normal group (*P* < 0.05). In mice treated with continuous Cy, the total follicle number (TF) of mice combined with VX-765 (C-Cy-Vx765) was higher than that of mice without VX-765, and the TF of the two groups was lower than that of the normal group (*P* < 0.05). The value of PMF/TF in C-Cy-Vx765 group was significantly higher than that in the other three groups, while that of EGF/TF was significantly lower (*P* < 0.05). Immunohistochemical results showed that the phosphorylated forms of the main proteins of the PI3K / PTEN / AKT pathway were found to be more positive in Cy treated mice. The Western blot analysis showed that when Cy and VX-765 were co-treated, the increased levels of these phosphorylated proteins decreased compared with those treated with Cy alone. The AMH level of infancy Cy and VX-765 co-treated mice was higher than that of infancy normal mice (*P* < 0.05). After the mice grew to sexual maturity, the AMH level of Cy and VX-765 co-treated mice was still higher than that of Cy treated mice (*P* < 0.05), and there was no significant difference with normal mice (*P* > 0.05).

**Conclusion:**

VX-765 can maintain the level of AMH and inhibit the recruitment of PMF, thus protecting mice from Cy induced gonadotropic toxicity. Accordingly, VX-765 may play a protective role in mice with ovarian injury caused by chemotherapy.

## 1 INTRODUCTION

Malignant tumors continue to remain a main global public health issue, which is the second leading cause of death in the United States [1]. Specifically, more than 35,000 women aged 15 to 39 are diagnosed with malignant tumors every year [2]. To date, chemotherapy remains the mainstay of treatment for malignant tumors. Commonly used cytotoxic chemotherapeutic drugs include alkylating agents, antimetabolic drugs, platinum, antibiotics, targeted drugs, and so forth. According to the toxicity of gonads of chemotherapeutic drugs, it can be divided into high-risk, medium risk and low-risk groups [3]. Alkylating agents have the strongest gonadal toxicity, which is represented by cyclophosphamide (Cy).

Chemotherapy can lead to ovarian follicle damage, matrix fibrosis and vascular damage in female patients, resulting in functional failure and eventual infertility [4]. Studies have shown that the incidence of ovarian function decline in women receiving alkylating agent chemotherapy can reach as high as 42% [5]. Moreover, the fertility of female patients with nasopharyngeal carcinoma was observed to decrease significantly following treatment, of which the natural pregnancy rate was significantly lower compared to patients with non-nasopharyngeal carcinoma [6]. Women receiving chemotherapy may develop osteoporosis [7], cardiovascular disease [8] and depression [9], which can affect their quality of life. In the past 40 years, due to strides made in multi-disciplinary comprehensive treatment schemes for patients suffering from malignant tumors, especially chemotherapy schemes, the survival rate has been greatly improved in such patients [10], thereby enabling the malignant tumor population of young women to rise daily with an increased survival time. This group can be expected to maintain their fertility or have restored endocrine function following successful malignant tumor treatment. Therefore, focusing on the ovarian damage caused by chemotherapy in women of childbearing age is vital in order to protect their fertility and improve their quality of life.

At present, the operations and measures to protect the fertility of young women with malignant tumors mainly include: ovarian transplantation or displacement, frozen oocytes or ovarian tissue, embryo cryopreservation, ovarian protective drugs, etc. Among them, the ovarian protection drug schemes reported in the literature include Gonadotropin-Releasing Hormone analogs (GnRH-a) [11], anti-Müllerianhormone (AMH) [12], melatonin [13], *etc*. However, there are still many arguments about the impact of these drugs on ovarian function and protection of the ovary. In addition, the growth of follicles before puberty does not depend on pituitary derived gonadotropins [14]. For young patients at this age, this ovarian protection strategy to inhibit the endocrine activities of gonads is not beneficial to the prevention of ovarian reserve consumption.

So far, the natural products reported in the literature include oral oyster polypeptides [15], curcumin [16], *etc*. These natural ingredients have a protective effect on premature ovarian failure in mice, while soy isoflavones [17] can protect the ovaries of middle-aged female rats from oxidative stress and diminish apoptosis. Hyaluronic acid - modified drug - loaded polyethylenimine - stearic acid, a special structure of targeting nanoparticles co-delivery with curcumin and paclitaxel, can increase the anti-tumor efficacy without increasing the adverse reactions as a promising strategy for therapy ovarian cancer [18]. However, there is no literature on nanoparticles in ovarian protection, and this new field needs to be further explored in the future.

In previous studies, it was found that mice treated with Caspase-1 inhibitor *in vivo* reduced the loss of primordial follicles induced by cyclophosphamide as well as the apoptosis of granulosa cells of growing follicles [19, 20] *via* inhibition of the PI3K / PTEN / AKT pathway [21]. VX-765 is a prodrug that is absorbed orally, which is then rapidly metabolized into the active metabolite vrt-043198 by a nonspecific esterase and is a selective inhibitor of Caspase-1, with significant inhibition effect of Caspase-1 and Caspase-4 [22]. At present, VX-765 has passed the clinical phase I safety evaluation of the U.S. Food and Drug Administration (FDA) and has been approved for a number of phase II clinical trials for the treatment of psoriasis (ClinicalTrials.gov Identifier: NCT00205465) and epilepsy (ClinicalTrials.gov Identifier: NCT01048255 and NCT01501383).

This study attempts to analyze follicle development in mice in conjunction with the main proteins of the PI3K / PTEN / AKT pathway by pretreating mice with VX-765 suffering from ovarian injury caused by cyclophosphamide chemotherapy and the serum AMH of infancy female mice after chemotherapy and at sexual maturity was detected by ELISA. Accordingly, this study further seeks to determine whether Caspase-1 inhibitors can protect against ovarian injury due to cyclophosphamide chemotherapy. Furthermore, it provides novel clinical insight into the recovery of endocrine function as well as the protection of fertility in young women undergoing chemotherapy following successful malignant tumor treatment.

## 2 MATERIALS AND METHODS

### 2.1 Experimental Animals

Female C57BL/6J mice (n = 50) (six-week-old; Gempharmatech-GD, China), before the experiment, were randomly divided into 5 groups (n = 10 each): single Cy with VX-765 group (S-Cy-Vx765), continuous Cy with VX-765 group (C-Cy-Vx765), single Cy chemotherapy group (S-Cy), continuous Cy chemotherapy group (C-Cy) and normal control group. Female C57BL/6J mice (n = 30) (12-day-old; Gempharmatech-GD, China), before the experiment, were randomly divided into 3 groups (n = 10 each): infancy single Cy with VX-765 group (i-S-Cy-Vx765), infancy single Cy chemotherapy group (i-S-Cy) and infancy normal control group. All mice were provided ad libitum access to food and water (deionized) and were housed at constant temperature (21±2°C), humidity (50%), and lighting (12-h light/dark, lights on at 7AM).

Procedures involving animals and their care were conducted in accordance with the ethically approved institutional guidelines and compliance with national and international laws and policies. The animal facility was approved (No. SYXK-2017-0081) by the Department of Science and Technology of Guangdong Province. All procedures were approved by the ethics committee of Sun Yat Sen University Laboratory Animal Center (Approval No. 2021000147).

### 2.2 Pharmacological Experiments

VX-765 (Belnacasan, HY-13205; MedChemExpress, China) 100mg/kg was dissolved in 20% Cremophor EL (HY-Y1890; MedChemExpress, China) and administered *via* gavage once a day for 21 consecutive days (S-Cy-Vx765, C-Cy-Vx765 and i-S-Cy-Vx765) (n= 30). The control mice (S-Cy, C-Cy, i-S-Cy and normal group) (n= 30) received an equal volume of the corresponding vehicle (physiological saline).

### 2.3 Mice Ovarian Injury Model

S-Cy-Vx765 and i-S-Cy-Vx765 animals were given a single intraperitoneal injection of 150 mg/kg of Cy (cyclophosphamide monohydrate, CS6029-Y; G-CLONE, China). C-Cy-Vx765 animals were given an intraperitoneal injection of 75 mg/kg of Cy once a week for 4 times. The Cy method of the S-Cy, C-Cy and i-S-Cy was the same as that of the S-Cy-Vx765, C-Cy-Vx765 and i-S-Cy-Vx765 groups. Normal group animals were given a single intraperitoneal injection of an equal volume of the vehicle (PBS).

All Cy usage commenced one a week after the final gavage of VX-765. All groups of animals were then humanely euthanized 7 d after the last Cy treatment, and their ovaries were subsequently removed for histologic or Western blot analysis.

### 2.4 Follicle Count

In this study, follicle counts at all levels were performed on the whole ovary. Ovaries were fixed in Bouin's Fluid (RS4140; G-CLONE, China) and were then embedded in paraffin in an automated tissue processor. Whole ovaries were then serially cut (every 5 µm) into 4 mm sections. One of the five sections was pasted onto the glass slides and stained with hematoxylin and eosin.

Follicle stage was classified according to accepted definitions and was then counted according to the following [23]: 1) primordial follicles (PMF), a nucleus surrounded by a single layer of flattened squamous follicular cells; 2) primary follicle, an oocyte surrounded by a single layer of cuboidal granulosa cells; 3) secondary follicle, 2 or more layers of cuboidal granulosa cells without the antrum; 4) antral follicles (AF), follicle with an antral cavity regardless of size; and 5) corpus luteum (CL), luteal cells that were approximately spherical in shape. A follicle was counted only if the nucleolus was identified in order to avoid counting the same follicle several times. PMFs were considered as resting follicles, whereas both primary and secondary follicles were classified as early growing follicles (EGF). Total follicles (TF) were regarded as the sum of PMF, EGF and AF.

### 2.5 Immunohistochemistry

Ovaries were dissected and fixed in 4% paraformaldehyde (PFA, PH0427; Phygene, China), washed, embedded in paraffin, and sectioned. The ovaries were cut into thick 4 mm sections and pasted onto the glass slides. The sections were then deparaffinized and rehydrated. Following epitope retrieval treatment (at 98°C for 30 min in 0.01 M citrate buffer solution, pH 6.0; C885556, Macklin, China), nonspecific binding sites were blocked for 1 h using blocking solution (Protein Block Serum Free) (BB-3541; BestBio, China) at room temperature. The sections were then incubated with appropriate primary antibodies antibody diluent at 4°C overnight. They were then washed in PBS and incubated for 1 h with appropriate secondary antibodies diluted in the same buffer. All sections were rinsed in PBS for 5 min, air dried, and cover-slipped using Glycerol Jelly Mounting Medium (BB-2347; BestBio, China). The tissue sections were then examined by microscopy. Image-Pro Plus software was used to analyze the data of the immunohistochemical pictures.

### 2.6 Western Blot Analysis

Ovaries were homogenized in a lysis buffer (RIPA lysis buffer; Abcam, China + protease and phosphatase inhibitors; Abcam, China) using a TissueLyser LT homogenizer (Qiagen, Germantown, MD, USA). They were then centrifuged 14000 g at 4°C for 30 min, after which the supernatant containing protein was taken and 5x loading buffer (Thermo Fisher, China) was added. It was then boiled using boiling water for 15 minutes so as to fully denature the protein. After SDS-PAGE was configured, the proteins were electrotransferred onto a PVDF. The membrane was then rinsed in TBS/Tween (EpiZyme, China) and treated with protein free rapid blocking buffer (EpiZyme, China). The membrane was incubated overnight at 4°C with the relevant antibodies. After incubation with horseradish peroxidase-labelled secondary antibody, the immunoreactivity was visualized with enhanced chemiluminescence (Omni-ECL™Femto Light Chemiluminescence Kit; EpiZyme, China). Membranes were then scanned with the Odyssey imaging system (LI-COR, Lincoln, NE, USA), and quantification was conducted using Image Studio 4.0 (LI-COR). All Western blot analyses represented 3 to 5 experiments. In this study, Image J was used to quantify the gray value of Western blots bands for analysis.

### 2.7 ELISA

The time points of blood collection were 1 week after a single intraperitoneal injection of Cy and when the mice grew to 8 weeks old. The collected blood of mice was allowed to stand for 1 hour, and the serum was separated after centrifugation at low speed. The concentrations of AMH were measured by enzyme-linked immunosorbent assay kit (Abcam, Shanghai, China).

### 2.8 Antibodies

FOXO3A (12829S; Cell Signaling Technology), phospho-FOXO3A (9464S; Cell Signaling Technology), mTOR (2983S; Cell Signaling Technology), phospho-mTOR (ab109268; Abcam), RPS6 (2211S; Cell Signaling Technology), phospho-RPS6 (ab225676; Abcam), AKT (4691S; Cell Signaling Technology), and phospho-AKT (ab38449; Abcam) were used for Western blot analysis and immunohistochemistry.

### 2.9 Statistical Analysis

All results were expressed as means ± SEM. Statistical differences between groups were tested using 1-way ANOVA and Kruskal-Wallis tests. Statistical analysis was performed *via* SPSS for Windows (SPSS, Inc., Chicago, IL, USA). A p-value < 0.05 was considered to be statistically significant.

## 3 RESULTS

### 3.1 Impact of Cy on Mice Ovaries

In order to evaluate the effect of Cy on the ovary, a model of ovarian injury following chemotherapy in mice was constructed. Six-week-old adolescent mice received a single intraperitoneal injection of Cy, a continuous intraperitoneal injection of Cy or the vehicle, respectively. The exhaustive PMF, EGF, AF and CL classes were then quantified for each group. In regard to follicle count, the number of follicles at all levels in S-Cy and C-Cy groups was found to be less than that in the normal group (*P <* 0.05) (Table **1**). In terms of follicle ratio, PMF/TF and EGF/TF were lower than that of the normal group (*P <* 0.05); however, no difference in AF/TF was observed (*P >* 0.05) (Table **2**). Therefore, Cy was inferred to have caused the consumption of the mice follicular pool. Accordingly, damage to the mice ovary was certain, and the animal model of ovarian damage due to chemotherapy was thus successfully established.

### 3.2 VX-765 Treatment Reduces Follicles Depletion induced by Cy

The impact of intraperitoneal injection of Cy on the ovaries, as well as the effect of coadministration of Cy and VX-765 on the follicle pool, were studied in pubertal female mice. Six-week-old mice were treated with a single or continuous intraperitoneal injection of Cy with or without VX-765 and were then compared to pubertal mice receiving the vehicle. Ovaries were removed 1 week after the last Cy treatment and were prepared for subsequent histological analysis.

The consumption of the follicle pool by Cy was confirmed, and mice treated with VX-765 prior to receiving Cy treatment (S-Cy-Vx765 and C-Cy-Vx765) were found to have more PMF than mice treated with Cy alone (S-Cy and C-Cy) (*P <* 0.05). In regard to EGF and AF, no difference among the four experimental groups (*P >* 0.05) was noted. However, their values were found to be lower than those in the normal group (*P <* 0.05). In TF, C-Cy-Vx765 was noted to be larger than C-Cy, and the TF of both groups was less than that of the normal group (*P <* 0.05). However, no difference was observed between the S-Cy-Vx765 and S-Cy groups (*P* > 0.05). For CL, normal grou*p >* S-Cy > S-Cy-Vx765 (*P* > 0.05) (Table **1**).

In terms of ratio, the value of PMF/TF in the C-Cy-Vx765 group was shown to be significantly higher than that in the other three groups (S-Cy-Vx765, S-Cy and C-Cy), while that of EGF/TF was significantly lower (*P <* 0.05). Meanwhile, PMF, EGF, AF, PMF/TF and EGF/TF in the normal group were found to be greater than those in the other four experimental groups. There was no significant difference in AF/TF value among the groups (*P* > 0.05) (Table **2**).

### 3.3 Effect of VX-765 on the PI3K / PTEN / AKT Pathway: Histological and Biochemical Analyses

In order to investigate the effect of VX-765 on the PI3K / PTEN / AKT signaling pathway, immunohistochemical and Western blot analyses were conducted using the ovaries from each group of mice treated with VX-765 or its vehicle. Following only Cy treatment or co-treatment with VX-765, the immunohistochemical expression of FOXO3a, mTOR, RPS6 and AKT, as well as its corresponding phosphorylated proteins in the follicles, are shown in Fig. (**1A**-**1D**) and Table **3**. Here, the phosphorylated forms of the PI3K / PTEN / AKT pathway proteins (FOXO3a, mTOR, RPS6 and AKT) were found to be more positive in Cy treated mice (S-Cy and C-Cy). According to the Western blot analysis, increased phosphorylation of FOXO3A, mTOR, RPS6 and AKT after Cy treatment alone was confirmed (S-Cy and C-Cy) [2]. When Cy and VX-765 were co-treated (S-Cy-Vx765 and C-Cy-Vx765), the increased levels of these phosphorylated proteins decreased compared with those treated with Cy alone (Fig. **S2A**, **S2B,** Supplementary figure).

### 3.4 Serum AMH Level in Infancy Chemotherapy Mice and After Sexual Maturity

**Fig. (3):**
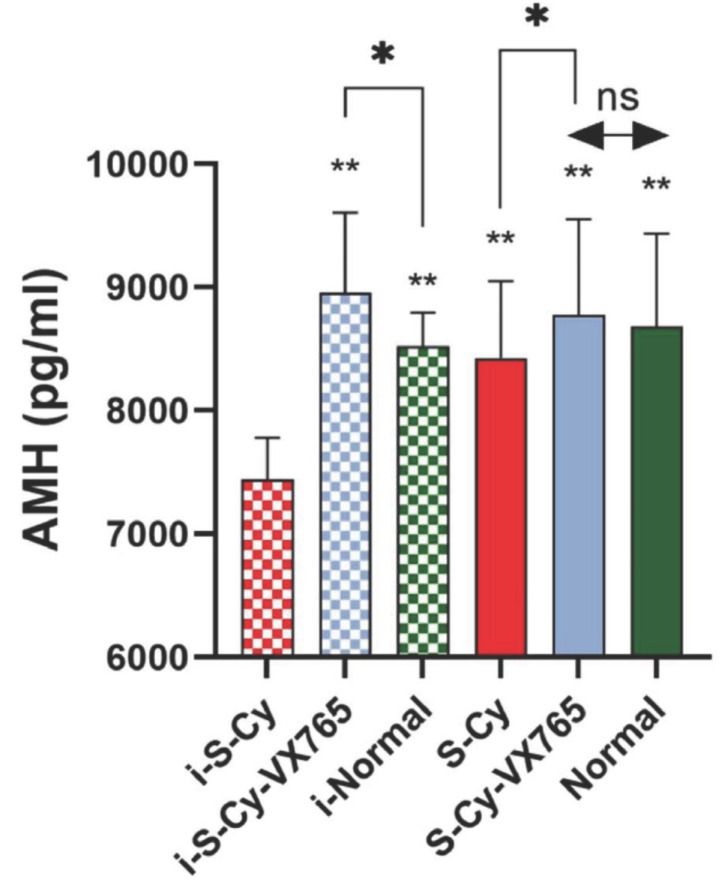
Serum AMH level in infancy chemotherapy mice and after sexual maturity. **: Compared with i-S-Cy group, *P <* 0.01; *: Comparison between groups, *P <* 0.05.

In the results of serum AMH, compared with i-S-Cy group, AMH in other groups was higher (*P <* 0.01). The AMH of i-S-Cy-Vx765 was higher than that of i-Normal (*P <* 0.05). The AMH of S-Cy-Vx765 was higher than that of S-Cy (*P <* 0.05). There was no significant difference in AMH level between S-Cy-Vx765 and Normal group (*P* > 0.05) (Fig. **3**).

## 4 DISCUSSION

Cy is mainly used for the treatment of malignant tumors, however, its particularly high gonadal toxicity continues to be a major issue. The use of gonadal toxic chemotherapeutic drugs represented by Cy was shown to directly act on division active EGF and cause a large number of deaths in the growing follicles. Afterward, the secretion of AMH (Anti-Müllerian hormone) was observed to decrease, the inhibition of PMF recruitment was relieved, and the PI3K / PTEN / AKT pathway was activated. At this time, a large number of PMFs accelerated their recruitment into growth and development and became split active EGF, further accelerating the consumption of PMF and disturbing the dynamic balance between PMF and EGF, which resulted in ovarian reserve decline [19, 20]. Indeed, Cy has been previously shown to induce the depletion of follicular reserves in a dose- and age-related manner in both women and mice [3, 24].

Embryo freezing, oocyte freezing and ovarian tissue freezing [25] serve as effective methods for fertility protection. However, as certain patients require urgent commencing of cancer treatment or due to economic reasons and dependence on assisted reproductive technology [6], these technologies have been subject to numerous restrictions in clinical applications, their biosafety has not been clearly defined, and they are unable to protect the endocrine function of the ovary. Currently, it is generally believed that gonadotrophin-releasing hormone agonist (GnRH-a) can protect ovarian reserves by inhibiting the gonadal axis [26, 27], 

In this study, the negative effect of Cy on PMF reserves was confirmed, which showed an increase in the EGF/TF ratio in the ovaries of Cy treated mice. These findings suggest an accelerated activation of PMF recruitment induced by Cy *via* upregulation of the ovarian PI3K / PTEN / AKT pathway. In addition, Cy can lead to follicle apoptosis, reducing the secretion of inhibitors that inhibit PMF activation [21, 12]. Gonadotoxicity, also known as the burnout effect, contrasts with the classic theory of a direct action of chemotherapy on oocyte DNA contained within the PMF, which result in apoptosis of these dormant follicles [19]. This study’s results were consistent with the burnout effect as the phosphorylation of important proteins in the PI3K pathway increased in Cy treated mice (S-Cy and C-Cy). In addition, the immunohistochemical results mirrored the results of the Western blot analysis. Other studies have also confirmed this mechanism in Cy as well as in other chemotherapeutic drugs [32-34].

The specific ICE/caspase-1 inhibitor VX-765 is a pro-drug with improved oral bioavailability developed for the treatment of inflammatory and autoimmune diseases [22, 35]. However, it has been sparsely used in reproductive system-related diseases. Since Caspase-1 inhibitor can inhibit the PI3K / PTEN / AKT pathway, and a number of phase II clinical trials are currently in progress, it was applied to mice undergoing chemotherapy in order to inhibit the activation of the PI3K / PTEN / AKT pathway for the Cy induced burnout effect. According to this study’s findings, the above hypothesis was verified. In mice co-treated with Cy and VX-765 (S-Cy-Vx765 and C-Cy-Vx765), PMF and TF were found to be more than those treated with Cy alone (S-Cy and C-Cy), regardless of whether Cy was used alone or continuously. In the C-Cy-Vx765 group, PMF/TF and EGF/TF were noted to be higher than S-Cy and C-Cy. Although PMF, EGF, AF, PMF/TF and EGF/TF of C-Cy-Vx765 did not reach the levels present in normal mice, the results suggested that mice using VX-765 had an inhibitory effect on follicular consumption induced by Cy treatment. According to immune though PMF is nongonadal hormone dependent [14]. Therefore, the ovarian protective effect of children with immature gonadal axes remains to be elucidated. The mechanism and clinical effectiveness of other ovarian protective drugs, such as inhibiting apoptosis and promoting angiogenesis [28-30], require further study. Some drugs that need to be administered directly to the ovary further limit their future clinical application [31]. Accordingly, there is an urgent need to find an effective method of fertility protection for female cancer patients of all ages. histochemistry, the phosphorylation expression of the PI3K / PTEN / AKT pathway proteins was also shown to be increased, while the phosphorylation forms of FOXO3A, mTOR, RPS6 and AKT were more positive in Cy treated mice. At the same time, the Western blot analysis generated consistent results in regard to the whole ovary. The phosphorylated forms of FOXO3A, mTOR, RPS6 and AKT were observed to be lower in S-Cy-Vx765 and C-Cy-Vx765 than in S-Cy and C-Cy. Although not all groups had significant differences in phosphorylated proteins, there were significant differences between C-Cy-Vx765 and C-Cy. This may be beneficial in clinical application as chemotherapy does not usually follow a single course of treatment. Overall, these results may provide novel evidence, confirm the negative effect of Cy on PMF reserves, and show the increase of EGF/TF ratio in the ovary of Cy treated mice in the present adolescent mouse model, indicating that VX-765 inhibits the recruitment of PMF and protects mice from Cy induced gonadotropic toxicity. However, the molecular mechanisms behind these effects have not been fully understood.

Interestingly, exploring the important proteins of the PI3K signaling pathway demonstrated that the phosphorylation levels of FOXO3A were significantly lower in the ovaries of VX-765 treated mice. FOXO3A is expressed in the nucleus of PMF and plays an important role in the maintenance of PMF in the dormant state [36]. This result was emphasized in FOXO3A -/- mice, which showed overall follicular activation, resulting in oocyte death and early consumption of ovarian reserves [34]. In addition, studies have shown that FOXO3A is located in the nucleus of PMF, and the phosphorylation of FOXO3A can induce protein nuclear output, leading to PMF activation [37]. Therefore, the phosphorylation levels of FOXO3A were found to be significantly lower following VX-765 treatment, suggesting that VX-765 may prevent the activation of PMF by preventing the cytoplasmic shuttle of FOXO3A during chemotherapy. However, the molecular mechanism behind how VX-765 plays a protective role in the ovary has yet to be fully understood and requires further investigation.

Regarding the dose of VX-765 that should be used in mice, this study is based on the human oral VX-765 dose of 300 mg TID provided in the clinical trial NCT01048255 completed in the United States in 2014. The age range of the included population is 18-64 years old. BMI value is 18-35kg/m2. The calculation is carried out according to the dose conversion formula between different types of animals in “Pharmacological Experiment Methods”. The specific calculation method is as follows: Convert 300mg TID to 900mg QD, and calculate the average weight of American adult males at 88kg and the average weight of women at 75kg. The dose is 10-10.23 mg/kg, the equivalent dose in mice is 120-122.76 mg/kg. Based on the literature of other animal experiments, the dosage of VX-765 is 50-200mg/kg, and the method of administration is either oral or intraperitoneal injection. Combined with the results of the death of pre-experimental mice after gavage with VX-765, the final decision was made to use 100mg/kg as the final dose. However, whether this is the optimal dose for ovarian protection remains to be explored in greater depth.

AMH is a glycoprotein secreted by ovarian granulosa cells, which reduces the depletion of a follicular pool by inhibiting the recruitment of primordial follicles. Because the level of serum AMH does not change with women's menstrual cycle, it is more stable than other sex hormones [38]. To predict the effect of chemotherapy exposure on ovarian function, the researchers evaluated the effect of AMH. The results showed that the serum AMH level decreased with the progress of chemotherapy. In addition, the serum AMH level of women who did not restore normal ovarian function was still low [39, 40]. This conclusion is similar to the results of this study. Among the results of serum AMH, infancy chemotherapy mice (i-S-Cy) had the lowest AMH (*P <* 0.01). Besides, the research data also show that women with higher AMH levels before and after chemotherapy are more likely to continue to maintain ovarian function [41]. It should be noted that in this study, the AMH level of infancy chemotherapy mice treated with VX-765 was even higher than that of infancy normal mice (*P <* 0.05). After the mice grew to sexual maturity, the AMH level of mice treated with vx765 (S-Cy-Vx765) was still higher than that of S-Cy (*P <* 0.05), and there was no significant difference between the AMH level of S-Cy-Vx765 and normal group (*P >* 0.05). These results suggest that VX-765 can inhibit the recruitment of PMF, which can play a role in infancy, and continuously protect the follicle pool during sexual maturation, which has an important impact on the maintenance of ovarian reserve.

Currently, in the field of oncofertility, efforts are being made to improve existing fertility preservation techniques and develop new strategies suitable for patients regardless of age and ovarian reserve status [42, 43].Thus, several “fertoprotectant” molecules, such as rapamycin or melatonin, have been developed and studied in order to limit chemotherapy induced ovarian damage in mice [44, 45]. However, these treatments involve the cell-death mechanism. Hence, they may interfere with the therapeutic efficacy of chemotherapy or the physiology of the reproductive system [31]. However, in order to be applied in clinical practice, these drugs should not reduce the anticancer activity of chemotherapeutic drugs or retain genetically defective oocytes. VX-765 has a positive effect on PMF reserves through the inhibition of the phosphorylation of the PI3K / PTEN / AKT pathway proteins, which inhibit the recruitment of PMF and protect mice from Cy induced gonadotropic toxicity, and also has a protective effect in infancy mice. Currently, VX-765 is used to treat inflammatory and autoimmune diseases, and its safety has been determined in clinical trials for psoriasis and refractory epilepsy. This study’s results may support the use of VX-765 as an orally active drug in future clinical trials for the protection of chemotherapy-related ovarian injury.

## CONCLUSION

In conclusion, in the adolescent mouse model, the negative effect of Cy on PMF reserves was demonstrated, which showed that the EGF/TF ratio increased in Cy treated mice. Meanwhile, VX-765 was shown to inhibit the recruitment of PMF, thus protecting mice from Cy induced gonadotropic toxicity. In the infancy young mouse model, it was confirmed that VX-765 could maintain the level of AMH, protect the ovarian reserve and continue to affect during sexual maturation. These results suggest that VX-765 plays a protective role in mice with chemotherapy-induced ovarian injury.

## Figures and Tables

**Fig. (1):**
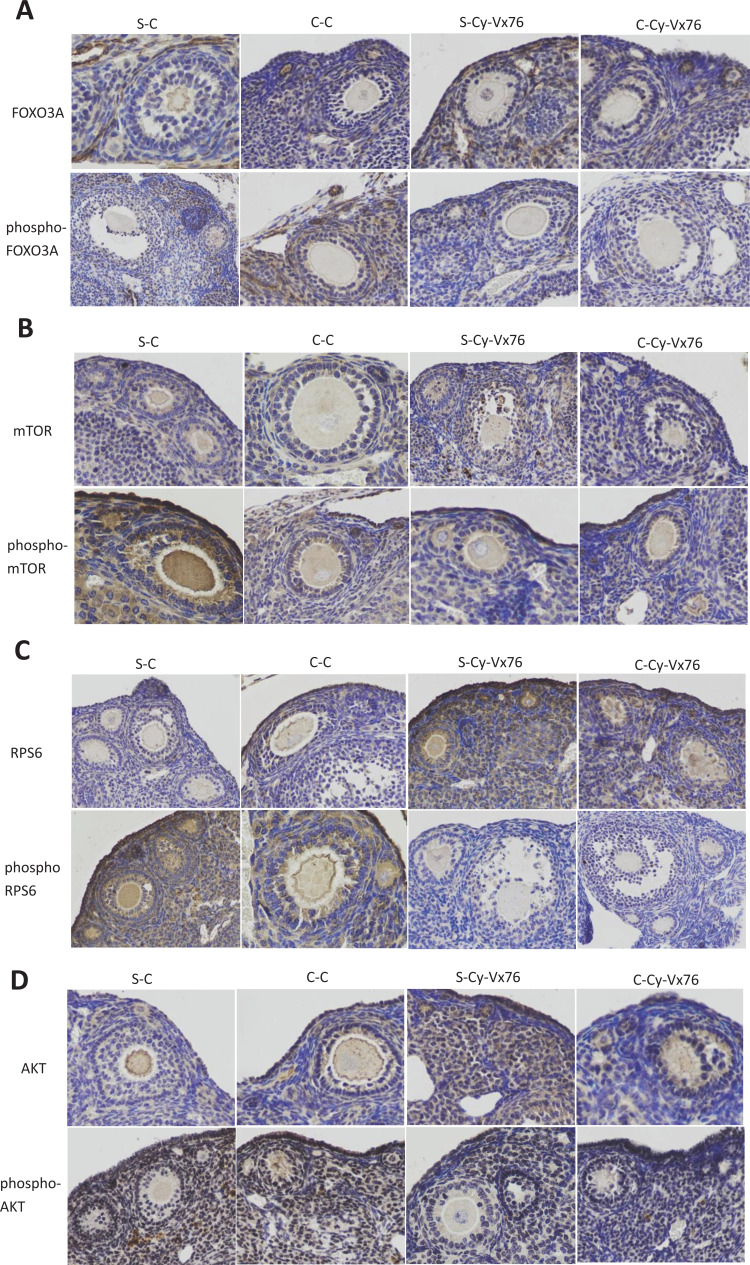
Effect of VX-765 on the PI3K / PTEN / AKT pathway. Panels A-D: Histologic analysis of ovarian sections 1 week after the last intraperitoneal injection of Cy. Representative photomicrographs showing changes in Cy-injection mice treated with the vehicle (S-Cy and C-Cy) or VX-765 (S-Cy-Vx765 and C-Cy-Vx765). **A**) FOXO3A and phospho-FOXO3A. **B**) mTOR and phospho-mTOR. **C**) RPS6 and phospho-RPS6. D) AKT and phospho-AKT. Panels E-F: Results of the Western blot analysis. *: *P <* 0.05; **: *P <* 0.01. S-Cy: single Cy group, C-Cy: continuous Cy group, S-Cy-Vx765: single Cy with VX-765 group, C-Cy-Vx765: continuous Cy with VX-765 group (see Materials and methods section for treatment paradigm).

**Fig. (2):**
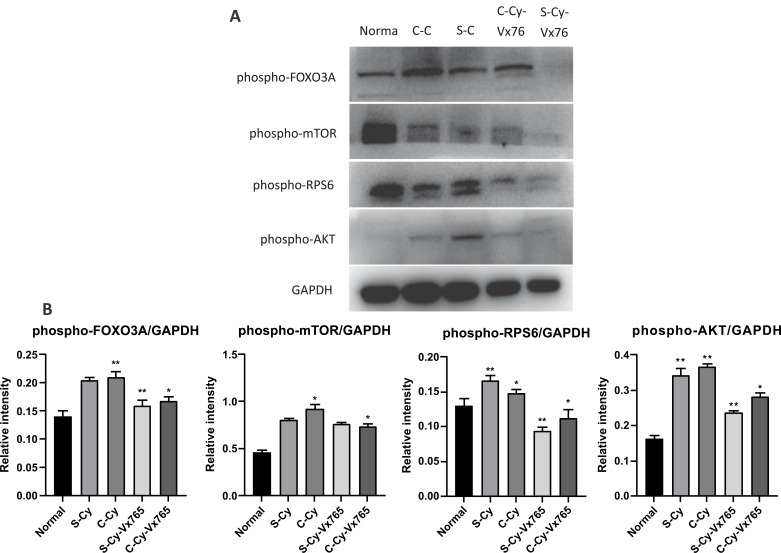
Effect of VX-765 on the PI3K / PTEN / AKT pathway. Panels **A, B**: Results of the Western blot analysis. **A**) Ovaries collected 1 week after the last injection were lysed and PI3K signaling proteins (phospho-FOXO3A, phospho-mTOR, phospho-RPS6 and phospho-AKT) were analyzed by Western blot analysis. Each sample was run in duplicate. **B**) Graph shows quantification of phosphorylated proteins in Graph shows quantification of phosphorylated proteins in PI3K / PTEN / AKT pathway. Graphs represent quantification of phospho-proteins from four independent experiments. *: *P <* 0.05; **: *P <* 0.01. S-Cy: single Cy group, C-Cy: continuous Cy group, S-Cy-Vx765: single Cy with VX-765 group, C-Cy-Vx765: continuous Cy with VX-765 group (see Materials and methods section for treatment paradigm).

**Table 1:** Follicles and corpus luteum counts of the mice in each group.

**-**	**PMF**	**EGF**	**AF**	**TF**	**CL**
S-Cy	8.25±1.389^a^	27.00±13.234^a^	3.63±1.923^a^	38.88±13.737^abc^	2.88±0.641^a^
C-Cy	8.38±3.096^a^	18.81±4.651^a^	2.25±0.931^a^	29.44±6.175^a^	1.82±0.750^b^
S-Cy-Vx765	12.38±2.560^b^	30.50±10.928^a^	2.75±0.463^a^	45.625±10.941^abc^	2.63±1.302^ab^
C-Cy-Vx765	21.00±2.582^c^	21.75±2.754^a^	4.00±1.155^a^	46.75±4.992^b^	2.50±1.732^abc^
Normal	35.12±2.561^d^	46.58±4.552^b^	7.54±1.584^b^	89.24±7.153^c^	5.43±9.492^c^
F value	123.023	11.404	18.807	8.079	13.341
P value	< 0.001	< 0.001	< 0.001	0.001	< 0.001

**Note:** Data were presented as mean (standard deviation) or N, unless otherwise stated. **Abbreviations:** PMF: primordial follicles, EGF: early growing follicles, AF: antral follicles, TF: total follicles, CL: corpus luteum. Superscript a, b, c or d, unless indicated differently, all represent statistical differences (*P <* 0.05).

**Table 2:** Ratio of PMF, EGF or AF to the total number of follicles was calculated for each group.

**-**	**PMF/TF**	**EGF/TF**	**AF/TF**
S-Cy	0.14±0.057^a^	0.40±0.035^a^	0.06±0.033
C-Cy	0.17±0.046^a^	0.39±0.026^a^	0.05±0.034
S-Cy-Vx765	0.18±0.060^a^	0.39±0.033^a^	0.04±0.011
C-Cy-Vx765	0.31±0.019^b^	0.32±0.013^b^	0.06±0.017
Normal	0.38±0.022^c^	0.50±0.023^c^	0.08±0.001
F value	26.784	24.661	1.723
P value	< 0.001	< 0.001	0.166

**Note:** Data were presented as mean (standard deviation) or N, unless otherwise stated. **Abbreviations:** PMF: Primordial follicles, EGF: early growing follicles, AF: antral follicles, TF: total follicles. Superscript a, b or c, unless indicated differently, all represent statistical differences (*P <* 0.05).

**Table 3:** Immunohistochemical results analysis data.

**-**	**Positive Cell**	**Positive Cell Density, ** **Number/ Number/10^4^pixels**	**Mean Density**	**H-Score**	**Positive Score**
**FOXO3A**					
S-Cy	45.132%	8.9654	0.2123	45.12	2
C-Cy	17.442%	4.4860	0.2108	17.24	1
S-Cy-Vx765	68.573%	14.7813	0.2203	68.57	3
C-Cy-Vx765	15.546%	3.4571	0.2073	15.46	1
*P* value	<0.001	<0.01	0.036	<0.01	<0.01
**Phospho-FOXO3A**					
S-Cy	74.381%	15.8993	0.2237	74.38	3
C-Cy	82.011%	17.3258	0.2314	82.14	4
S-Cy-Vx765	9.876%	1.3489	0.2008	9.76	1
C-Cy-Vx765	9.903%	1.3526	0.2037	9.90	1
*P* value	<0.001	<0.001	0.022	<0.001	<0.01
**mTOR**					
S-Cy	36.075%	7.5041	0.2143	36.07	2
C-Cy	70.638%	15.0375	0.2204	70.64	3
S-Cy-Vx765	34.084%	6.4138	0.2104	33.30	2
C-Cy-Vx765	35.468%	6.7886	0.2165	35.46	2
P value	<0.001	<0.001	0.028	<0.001	<0.01
**Phospho-mTOR**					
S-Cy	96.143%	19.0147	0.2393	96.14	4
C-Cy	79.432%	16.6470	0.2284	79.43	4
S-Cy-Vx765	41.378%	7.5041	0.2134	41.30	2
C-Cy-Vx765	43.643%	7.7987	0.2109	43.64	2
*P* value	<0.001	<0.001	0.033	<0.001	<0.001
**RPS6**					
S-Cy	13.458%	2.4569	0.2175	13.47	1
C-Cy	21.003%	2.7983	0.2203	20.36	1
S-Cy-Vx765	87.884%	16.6892	0.2387	87.95	4
C-Cy-Vx765	86.724%	16.7593	0.2996	86.72	4
*P* value	<0.001	<0.001	0.01	<0.001	<0.001
**Phospho-RPS6**					
S-Cy	96.034%	18.0476	0.2354	96.03	4
C-Cy	95.023%	17.7621	0.2321	95.67	4
S-Cy-Vx765	8.684%	1.6958	0.2082	8.68	1
C-Cy-Vx765	7.925%	1.6500	0.2101	7.87	1
*P* value	<0.001	<0.001	0.024	<0.001	<0.001
**AKT**					
S-Cy	11.865%	2.4398	0.2076	11.76	1
C-Cy	9.796%	1.7102	0.2149	9.80	1
S-Cy-Vx765	79.482%	16.7843	0.2298	78.53	4
C-Cy-Vx765	13.891%	2.5796	0.2086	13.89	1
*P* value	<0.001	<0.001	0.035	<0.001	<0.001
**Phospho-AKT**					
S-Cy	88.374%	17.3443	0.2319	89.94	4
C-Cy	93.274%	17.7861	0.2311	93.27	4
S-Cy-Vx765	48.000%	8.3800	0.2134	48.00	2
C-Cy-Vx765	45.462%	7.9875	0.2109	45.36	2
*P* value	<0.001	<0.001	0.016	<0.001	<0.01

## Data Availability

The analysed data for the current study will be available from the corresponding author.

## LIST OF ABBREVIATIONS

GnRH-a: Gonadotropin-Releasing Hormone analogs
AMH: SCoV-SG; PDB: 6VYB) anti-Müllerianhormone
MDA: Malondialdehyde
