# Supplementary material for: VX-765 has a Protective Effect on Mice with Ovarian Injury Caused by Chemotherapy
Source: Curr Cancer Drug Targets. 2023 Jan 11;23(4):307–18. doi: 10.2174/1568009622666220930110024 (PMC10202083; doi:10.2174/1568009622666220930110024)
Supplement: Supplementary file 1 [file CCDT-23-307_SD1.pdf]

Supplementary Materials

VX-765 has a Protective Effect on Mice with Ovarian Injury Caused by Chemotherapy

Pingyin Lee[1,2], Canquan Zhou[1,2] and Xiaokun Hu[1,2,*]

[1]Reproductive Medicine Center, The First Affiliated Hospital of Sun Yat-sen University, Guangzhou, China; [2]Guangdong Provincial Key Laboratory of Reproductive Medicine, The First Affiliated Hospital of Sun Yat-sen University, Guangzhou, China

SUPPLEMENTARY FIGURES

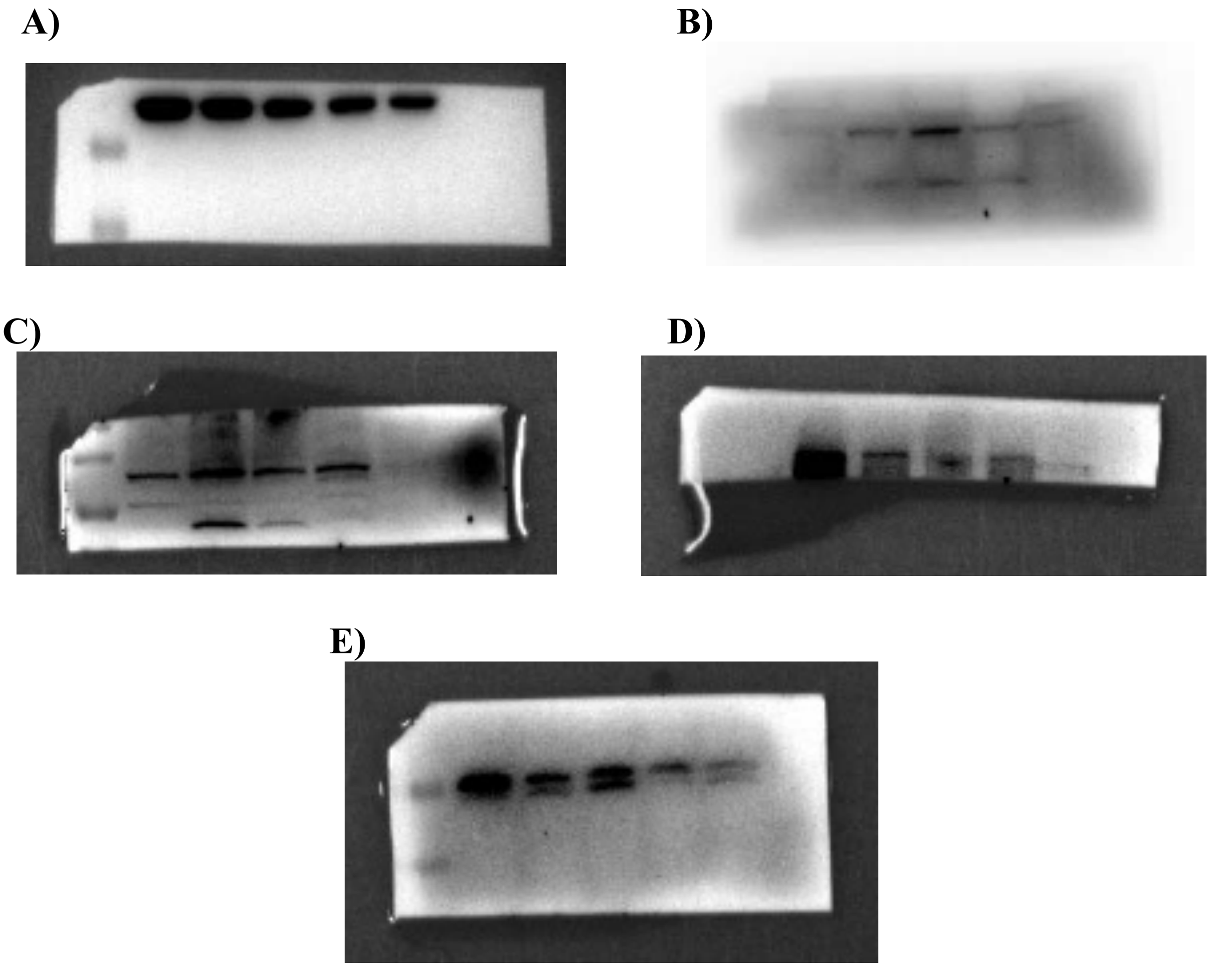

Supplementary Fig. S1. The ovaries collected one week after the last injection were lysed, and PI3K signal proteins (phosphate FOXO3A, phosphate mTOR, phosphate RPS6 and phosphate AKT) were analyzed by Western blot. Each sample is run repeatedly. The figure shows the uncropped gels of all Western blot analysis results. **A**) GAPDH, **B**) phospho-AKT, **C**) phospho-FOXO3A, **D**) phospho-mTOR, **E**) phospho-RPS6.

1873-5576/23 **© 2023 Bentham Science Publishers**
